# Nonlinearities in shadowgraphy experiments on non-equilibrium fluctuations in polymer solutions

**DOI:** 10.1140/epje/s10189-022-00195-1

**Published:** 2022-04-26

**Authors:** D. Zapf, J. Kantelhardt, W. Köhler

**Affiliations:** Physikalisches Institut, Universität Bayreuth, 95440 Bayreuth, Germany

## Abstract

**Abstract:**

Giant thermal and solutal non-equilibrium fluctuations are observed in shadowgraphy experiments on liquid mixtures subjected to a temperature gradient. For large temperature differences, both the temperature and the composition dependence of the relevant thermophysical parameters and the nonlinear terms in the diffusion equation need to be taken into account, leading to a nonlinear concentration profile. For temperature differences exceeding the inverse of the Soret coefficient, in our example approximately 10 K, the usual data evaluation yields increasingly wrong diffusion and Soret coefficients that are off by almost a factor of two for a temperature difference of 50 K. A local model that treats the measured shadowgraph signal as a superposition of the contributions from every layer of the sample is able to capture the essential trend and yields a good agreement with experimental data. The results are important for the application of shadowgraphy as a tool for the measurement of Soret and diffusion coefficients, where large temperature gradients promise a good signal-to-noise ratio.

**Graphical abstract:**

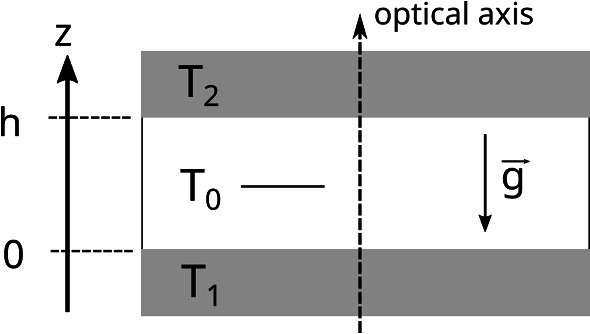

## Introduction

Thermodynamic non-equilibrium fluctuations (NEFs) are observed in liquids under the influence of a temperature or a concentration gradient [1, 2]. Their amplitude is proportional to the square of the respective gradient and diverges for small wavevectors $\sim q^{-4}$. In the absence of gravity, the growth of the amplitude is only limited by the finite sample size [3, 4]. Under gravity conditions, the amplitude levels off below a critical roll-off wavevector $q_{\mathrm{ro}}$ due to buoyancy and sedimentation [5].

Shadowgraphy (SG) has become the method of choice for the investigation of NEFs, and the technique has been turned into an analytical tool for the measurement of thermal diffusivities and diffusion and Soret coefficients [6–15]. When a binary mixture is subjected to a temperature gradient, the Soret effect leads to a superimposed concentration gradient and both thermal and solutal NEFs can simultaneously be observed. For wavevectors larger than the thermal roll-off $q_{\mathrm{ro}}^T$, the thermal diffusivity $D_{\mathrm{th}}=1/(\tau ^T q^2)$ follows directly from the *q*-dependence of the correlation time $\tau ^T$ of the thermal NEFs and the Fickian diffusion coefficient $D=1/(\tau ^c q^2)$ from the correlation time $\tau ^c$ of the solutal ones for wave vectors larger than the concentration roll-off $q_{\mathrm{ro}}^c$. The Soret coefficient is defined by the ratio of the concentration and the temperature gradient, which both can be determined from the amplitudes of the respective NEFs or, more conveniently, from the critical roll-off wavevectors $q_{\mathrm{ro}}^T$ and $q_{\mathrm{ro}}^c$ [16]. In particular, the latter possibility distinguishes shadowgraphy from other optical techniques, where concentration and temperature changes are more directly measured via associated refractive index changes.

Measurements of Soret and thermodiffusion coefficients require nonequilibrium conditions with a temperature gradient. As a rule, a larger temperature gradient translates to a better signal with less noise. But how much is too much? For most techniques, the temperature differences are relatively small: below one mK for thermal diffusion forced Rayleigh scattering (TDFRS) [17–19], $1\,\hbox {K}$ for optical beam deflection (OBD) [20–22] and 5–10 K in the case of optical digital interferometry (ODI) in the laboratory [23] or during the DCMIX project aboard the International Space Station [24, 25].

Common to all experimental techniques is the usual assumption that the experiments are essentially performed at the mean sample temperature, that deviations from equilibrium are small, and that temperature and composition dependences of system parameters and transport coefficients are negligible. In this contribution, we will go beyond this linear model and investigate the consequences of various nonlinearities for the investigation of NEFs by means of the shadowgraphy technique with large temperature gradients. We will take both the temperature dependence of important physicochemical parameters and nonlinear concentration profiles that result from the nonlinear thermodiffusion equation into account. The results of this paper are particularly important for the application of shadowgraphy as a tool for the measurement of diffusion, thermodiffusion and Soret coefficients.

The work presented here has been performed within the framework of the Giant Fluctuations (NEUF-DIX) project of ESA, which aims at the investigation of NEFs in complex multicomponent mixtures under microgravity conditions, and the associated CORA-MAP project ‘Technologies for Non-Equilibrium Systems’ (TechNES) for the development of NEFs into diagnostic tools. One major objective of these projects is the investigation of non-ideal systems, including the case of large gradients that give rise to nonlinearities and preclude the analytical modeling of the system by means of linearized hydrodynamics [26, 27].

## Experiment

**Fig. 1:**
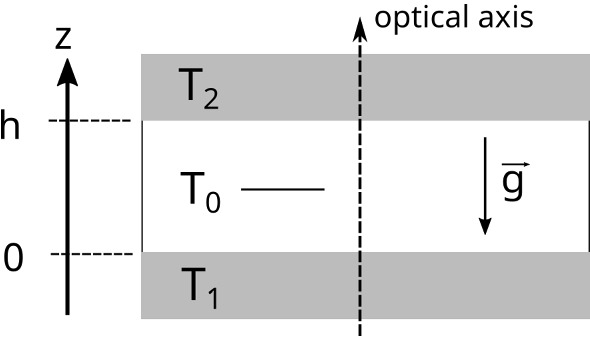
Sketch of the Soret cell for shadowgraphy experiments. The sample is enclosed between two horizontal sapphire windows with a distance *h* kept at temperatures $T_1$ and $T_2$. The optical axis of the detection light beam is oriented anti-parallel to the gravitational acceleration $\mathbf {g}$. Even for large temperature differences $\varDelta T = T_2 - T_1$, the mean sample temperature $T_0 = T_1 + \varDelta T /2$ is close to the midplane of the cell

We have used the identical shadowgraphy setup as described in Ref. [16]. It is built around a Soret cell with a vertical temperature gradient between two sapphire plates, whose inner surfaces are located at $z=0$ and $z=h$. Their temperatures are kept at $T_1 = T(z=0) = T_0 - \varDelta T/2$ and $T_2 = T(z=h) = T_0 + \varDelta T/2$, respectively. The thickness of the fluid layer is $h=5\,\hbox {mm}$. The thermal conductivity is approximately constant (see below) throughout the sample and the mean sample temperature $T_0$ is assumed close to the midplane of the cell (Fig. 1).

The collimated beam of a superluminescent diode (Superlum SLD, $\lambda = 670\,\hbox {nm}$) is used for illumination of the $13\,\hbox {mm}$ wide free aperture. The interference of the scattered and primary beams are detected by a CMOS camera (Hamamatsu Orca Fusion) at a distance $Z = 20.87\,\hbox {cm}$. The maximum employed acquisition rate without pixel binning is $50\,\hbox {Hz}$ for up to 200 images and $10\,\hbox {Hz}$ for up to 1100 images. A wider dynamic range was achieved by concatenating the structure functions acquired with different sampling rates, thereby ensuring a high sampling rate for the fast and a low sampling rate for slow processes within the same structure function. The temporal overlap of both series allows for a consistent amplitude normalization in the overlap time regime. The sample is a solution of polystyrene of $M_w = 17.9\,\hbox {kg/mol}$ with a polydispersity $M_w/M_n = 1.03$ (Polymer Standards Service GmbH) in toluene at a concentration of $c_0 = 0.01$ mass fractions.

## Results and discussion

The steady-state concentration distribution in a binary mixture of concentration *c* (mass fraction of first component) is determined by the Soret coefficient $S_T$ via

1 $$\begin{aligned} \nabla c = - S_T c(1-c) \nabla T \,, \end{aligned}$$

which follows directly from the cancellation of the Fickian diffusion and the thermodiffusion flows. In practically all Soret experiments, it is assumed that the Soret coefficient is constant, and concentration changes within the sample are small. In this case, the concentration term on the right-hand side of Eq. (1) can be approximated by the mean concentration $c_0$ and the constant concentration gradient is given by $\nabla c = - S_T c_0(1-c_0) \nabla T$. Measurements of the Soret coefficient at temperature $T_0$ and concentration $c_0$ require the determination of $\nabla c$ and the knowledge of $\nabla T$.

Although it is widely used, the validity of this linearized approximation needs to be questioned for two reasons. First, the nonlinear concentration term in Eq. (1) can only be taken as constant for small concentration changes, which are only to be expected in the case of small temperature differences $\varDelta T \ll S_T^{-1}$. Note that this criterion does not per se preclude strong temperature and concentration gradients as long as they act only over short distances. Secondly, the Soret coefficient $S_T(c,T)$ itself, as well as other system parameters, may depend on both concentration and temperature, which introduces yet another nonlinearity. Thus, in the general case of finite temperature differences, Eq. (1) holds only locally, the concentration gradient $\nabla c(x)$ becomes position dependent, and the concentration profile becomes nonlinear. In the following, we will discuss this situation.

### Temperature and concentration dependence of thermophysical parameters

The purpose of this section is to provide expressions for the temperature and concentration dependence of all relevant thermophysical parameters that will later be needed for the model calculations. Our goal is to keep it as simple as possible and to catch only the relevant dependencies, thereby ignoring the insignificant ones. Since we are dealing with dilute solutions, we will neglect the concentration dependence and consider only the temperature dependence of certain parameters of pure toluene. This is a reasonable approximation for coefficients that depend only weakly on concentration, such as the thermal diffusivity or the thermal conductivity.

*Soret coefficient* In order to solve Eq. (1) for arbitrary temperature gradients, the knowledge of both the concentration and temperature dependence of the Soret coefficient $S_T(c,T)$ is required. Such data for polymer solutions are hardly available, but for polystyrene in toluene, an empirical parameterization is derived in Ref. [28] on the basis of molar mass- and concentration-dependent measurements at the reference temperature $T_0 = 298.15\,\hbox {K}$ taken from Ref. [29]:

2 $$\begin{aligned} S_T(c,T)= & {} S_T(c,T_0) \left( \frac{T_0}{T} \right) ^{2.4}\nonumber \\ S_T(c,T_0)= & {} \frac{a}{1 + b\, c\,^\beta } \nonumber \\ a= & {} 3.294\times 10^{-4}\, M^{0.58}\nonumber \\ \beta= & {} 35.42\, M^{-0.5} + 0.82\nonumber \\ b= & {} \frac{a}{0.012} - 1 \end{aligned}$$

**Fig. 2:**
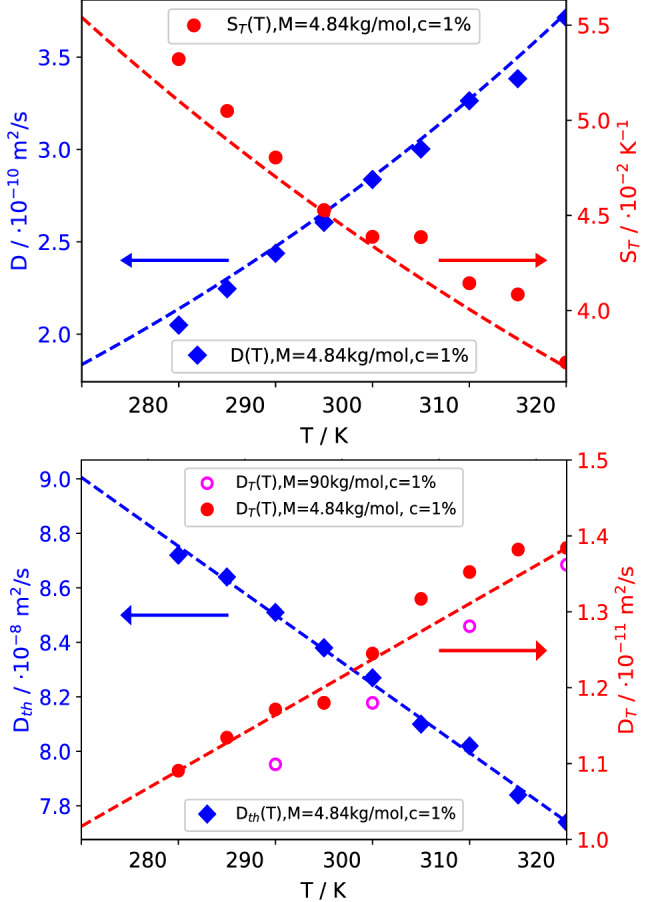
Temperature dependence of the Soret coefficient $S_T$ and the diffusion coefficient *D* (top) and of the thermal diffusivity $D_{\mathrm{th}}$ and the thermodiffusion coefficient $D_T$ (bottom) of PS ($M=4.8\,\hbox {kg/mol}$, $c=0.01$) in toluene as measured by TDFRS. The dashed curve for $S_T$ is calculated from Eq. (2) and the one for $D = D_T/S_T$ from Eqs. (2)–(3). $D_T$ is fitted by Eq. (3) and $D_{\mathrm{th}}$ by Eq. (4). $D_T$-data for $M=90\,\hbox {kg/mol}$, $c=0.01$ from Ref. [30] are shown for comparison

*M* is the molar mass in g/mol. While the concentration and molar mass dependence in Eq. (2) are based on experiments over a broad molar mass and concentration range, the temperature dependence was only measured for a single relatively high molar mass of $M = 90\,\hbox {kg/mol}$ and then assumed to hold also for all other molar masses. In order to verify its applicability also for significantly lower molar masses, we have measured the temperature dependence of $S_T(c=0.01,T)$ for $M = 4.8\,\hbox {kg/mol}$, which contains just below 50 repeat units and barely reaches the polymer limit of long chains, by means of the Thermal Diffusion Forced Rayleigh Scattering (TDFRS) technique [17, 18]. As shown in Fig. 2, the parameterization of the temperature dependence by Eq. (2), although not perfect, still gives a good description even at this low-*M* limit of the parameter range.

*Thermodiffusion coefficient and diffusion coefficient* A parameterization of the diffusion coefficient *D*(*c*, *T*) can be obtained according to $D = D_T/S_T$ with $S_T(c,T)$ from Eq. (2). The thermodiffusion coefficient $D_T$ is molar mass independent for chains exceeding the Kuhn segment of approximately $1\,\hbox {kg/mol}$ and its composition dependence can be neglected as long as *c* stays below about ten percent [30]. Its temperature dependence is only weak and the $D_T$ data from the same temperature-dependent TDFRS measurement as above can be fitted by

3 $$\begin{aligned} D_T= & {} [1.20 + 0.73 \times 10^{-2} (T-T_0)] \times 10^{-11}{\frac{\mathrm{m}^2}{\mathrm{sK}}}~, \end{aligned}$$

again with the reference temperature $T_0 = 298.15\,\hbox {K}$. The experimental data with the fit are plotted in Fig. 2.

Also shown for comparison are the only available other temperature-dependent measurements from Ref. [30] for $M = 90\,\hbox {kg/mol}$, which confirm the molar mass independence of $D_T(T)$. Thus, Eq. (2) provides a decent description for $S_T(c,T)$ over the entire relevant molar mass, composition and temperature range and, in combination with Eq. (3), also for *D*(*c*, *T*) for all molar masses and for polymer concentrations up to at least $c=0.1$. At even higher concentrations, $D_T$ begins to slow down due to the approaching glass transition. Since no temperature-dependent data exist for $c>0.1$, the parameterization of $D_T$ and, hence, also for *D*(*c*, *T*) is limited to $c < 0.1$. There is a strong need for new experimental data that would allow to extend the parameterization also to higher polymer concentrations. The experiments and simulations reported later were conducted for a molar mass of $M=17.9\,\hbox {kg/mol}$, but since we are currently also working with shorter chains, we have tested the validity of Eq. (2) down to the lower molar mass of $M=4.8\,\hbox {kg/mol}$.

*Thermal diffusivity* Figure 2 shows the temperature dependence of the thermal diffusivity $D_{\mathrm{th}}$ of the sample, which has also been measured by TDFRS. Neglecting its concentration dependence, it is parameterized by

4 $$\begin{aligned} D_{\mathrm{th}} = [8.3 - 2.5 \times 10^{-2} (T-T_0)] \times 10^{-8}{\frac{\hbox {m}^2}{\hbox {s}}}~, \end{aligned}$$

*Thermal conductivity* The temperature dependence of the thermal conductivity $\kappa (T)$ of toluene has been reported by Kashiwagi et al. [31]:

5 $$\begin{aligned} \kappa (T) = \left[ 0.1307 - 2.88 \times 10^{-4}{\hbox {K}^{-1}} \times (T-T_0) \right] {\frac{\hbox {W}}{\hbox {m}\,\hbox {K}}} \end{aligned}$$

We use this expression also for our moderately concentrated polymer solutions.

*Density and expansion coefficients* Densities $\rho (c,T)$ were measured by means of a vibrating tube density meter (Anton-Paar DSA 5000 M). Together with the temperature dependence of the density of pure toluene from Ref. [31], they lead to a parameterization

6 $$\begin{aligned} \rho (c,T) = \left[ 863 + 182 \, c - 0.933 \, (T-T_0) \right] {\frac{\hbox {kg}}{\hbox {m}^3}} ~. \end{aligned}$$

Since higher-order terms are very small and can be neglected, the thermal and the solutal expansion coefficients are evaluated for $c=0.01$ and $T=T_0$ and taken as constant (note that an erroneous value for $\beta _T$ is reported in Ref. [16]):

7 $$\begin{aligned} \beta _T= & {} \frac{1}{\rho }\left( \frac{\partial \rho }{\partial T} \right) _{p,c} = -1.08 \times 10^{-3}{\hbox {K}^{-1}} \end{aligned}$$

8 $$\begin{aligned} \beta _c= & {} \frac{1}{\rho }\left( \frac{\partial \rho }{\partial c} \right) _{p,T} = 0.210 \end{aligned}$$

*Viscosity* An empirical formula for the molar mass, concentration and temperature dependence of the dynamic viscosity of PS/Tol is given in Ref. [28]. It was, however, developed in particular for high polymer concentrations that are entangled and already approach the glass transition. It shows significant deviations at lower concentrations, where it does not describe the temperature dependence very well [28]. A better parameterization of the viscosity $\eta$ of a dilute to semidilute polymer solution in a solvent of viscosity $\eta _0$ can be based on the truncated Martin’s equation given in Ref. [32] for the specific viscosity $\eta _{sp} = \eta /\eta _0 -1$:

9 $$\begin{aligned} \eta _{sp} = \tilde{c}[\eta ] \left( 1 + K_H \tilde{c}[\eta ] + \frac{1}{2} (K_H \tilde{c}[\eta ])^2 + \frac{1}{6} (K_H \tilde{c}[\eta ])^3 \right) \end{aligned}$$

Here, $K_H \approx 0.4$ is the Huggins constant. The intrinsic viscosity $[\eta ]$ is measured in mL/g and the concentration $\tilde{c} = c \, \rho \times 10^{-3}$ has the matching units g/mL. Equation (9) gives a good description [32] up to at least $\tilde{c}[\eta ] = 2$.

The molar mass dependence of the intrinsic viscosity of a polymer in a given solvent is frequently described by a scaling law $[\eta ] = K M^a$, known as the Kuhn–Mark–Houwink–Sakurada or Staudinger–Kuhn equation. Wagner has reviewed this relation for atactic polystyrene in various solvents and found a perceptible deviation [33]. He suggests an improved parameterization

10 $$\begin{aligned} \log [\eta ] = -0.538 + 0.203 (\log M) + 0.0471 (\log M)^2 \end{aligned}$$

for $\eta$ in mL/g and *M* in g/mol. Equation (10) is valid over the entire molar mass range, at least from 660 to $4 \times 10^6\,{\hbox {g/mol}}$, and not very sensitive to temperature [33].

The last missing ingredient is the temperature dependence of the viscosity of toluene $\eta _0$, for which standard reference data are provided by Santos et al.[34] for a pressure of 0.1 MPa:

11 $$\begin{aligned} \ln \eta ^* = -5.2203 + \frac{8.964}{T^*} - \frac{5.834}{(T^*)^2} + \frac{2.089}{(T^*)^3} \end{aligned}$$

The reduced variables are $T^*=T/T_0$ and $\eta ^* = \eta _0(T) / \eta _0(T_0)$ with $T_0 = 298.15\,\hbox {K}$ and $\eta _0(T_0) = 554.2\,{\mu \hbox {Pa}\,\hbox {s}}$. Finally, the dynamic viscosity of the polymer solution is calculated from Eqs. (9), (10), and (11) as $\eta (c,T) = \eta _0(T)(\eta _{sp}(c) + 1)$. Together with Eq. (6), the kinematic viscosity $\nu (c,T) = \eta (c,T) / \rho (c,T)$ is obtained.

#### The temperature and concentration profiles

*Temperature profile* The temperature profile is not strictly linear because of the temperature dependence of the thermal conductivity $\kappa$ Eq. (5). The solution of the stationary heat equation

12 $$\begin{aligned} \nabla \cdot \left( \kappa (T) \nabla T \right) = 0 \end{aligned}$$

with a thermal conductivity that depends linearly on temperature, $\kappa (T) = k_0 + \alpha (T-T_0)$, is given by [35]

13 $$\begin{aligned} \frac{T(z) - T_1}{\varDelta T} = \frac{-1 + \left( 1 + 2 \beta \xi + \beta ^2 \xi \right) ^{1/2}}{\beta } ~, \end{aligned}$$

with $\beta = \alpha \varDelta T / k_0$, $\xi = z/h$, and $T_1 = T(z=0)$ being the temperature of the lower plate (Fig. 1). Equation (13) together with $\kappa (T)$ from Eq. (5) yields a temperature profile that is only slightly curved with a temperature gradient that deviates by less than $\pm 4$ percent from its mean value for a temperature difference of $\varDelta T = 30\,\hbox {K}$.

*Concentration profile  * Since $S_T(c,T)$ in Eq. (2) is a product of a concentration and a temperature-dependent term, Eq. (1) can be solved in its one-dimensional form after separation of variables to compute *c*(*z*) in the interval $0 \le z \le h$:

14 $$\begin{aligned} \int _{c(z=0)}^{c(z)} \frac{dc}{S_T(c,T_0) \,c(1-c)} = \int _0^z \nabla T(z) \left( \frac{T_0}{T(z)} \right) ^{2.4} \mathrm{d}z \end{aligned}$$

with the local temperature from Eq. (13). The integrals in Eq. (14) are computed numerically and the integration constant $c(z=0)$ is determined from the condition of mass conservation:

15 $$\begin{aligned} \int _0^h c(z) \mathrm{d}z = c_0 h \end{aligned}$$

**Fig. 3:**
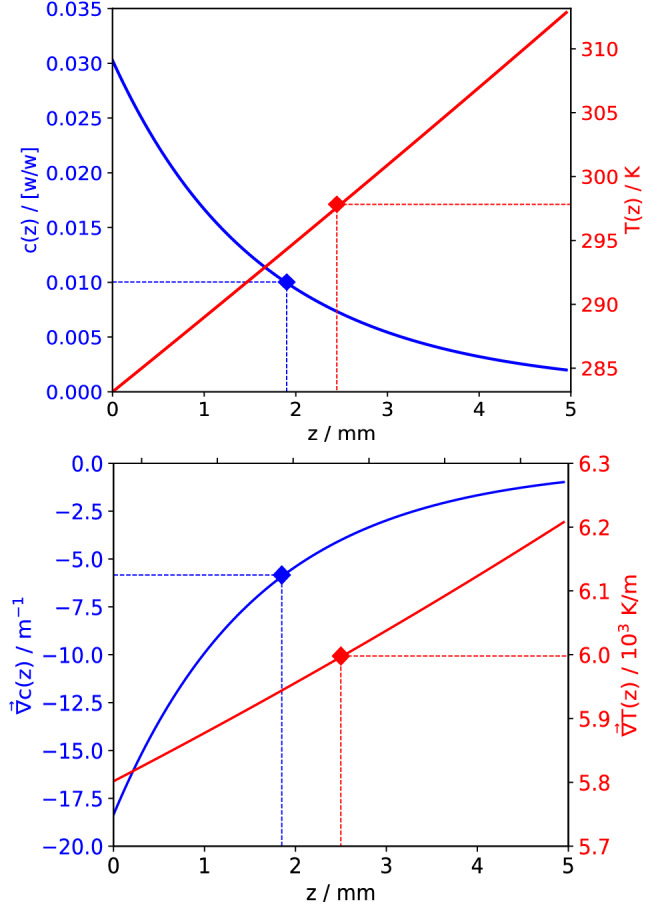
Concentration and temperature along the vertical axis (top) and corresponding gradients (bottom). The diamonds indicate the respective mean values, which are shifted away from the center of the cell particularly in the case of the nonlinear concentration profile. PS($17.9\,\hbox {kg/mol}$)/Tol, $c_0 = 0.01$, $T_0 = 298.15\,\hbox {K}$, $\varDelta T = 30\,\hbox {K}$

Figure 3 shows the stationary temperature and concentration profiles and the respective gradients obtained for a cell height of $h = 5\,\hbox {mm}$, a mean temperature of $T_0 = 298.15\,\hbox {K}$, a temperature difference of $\varDelta T = 30\,\hbox {K}$, and a mean concentration of $c_0 = 0.01$. Due to the pronounced nonlinearity of the concentration profile, the mean values of both *c* and $\nabla c$ are no longer observed in the center of the cell, but rather shifted toward the cold side. As a consequence, their positions do no longer coincide with the respective positions of *T* and $\nabla T$, which are still almost perfectly centered. For the case of temperature-independent thermophysical coefficients, where the nonlinearity stems solely from the nonlinear diffusion equation, the concentration distribution and gradient still look very similar, albeit with a reduced amplitude, in particular on the cold side.

#### The shadowgraphy experiment

In the following treatment of the shadowgraphy experiment and the differential dynamic analysis (DDA) [36–42], we closely follow our previous publication [16], albeit with a notation that is somewhat better adapted to the current literature [6].

The light scattered by NEFs in a thin liquid layer is recorded at a distance *Z* from the center of the layer by the sensor of the camera. Starting point is the structure function of Fourier transformed difference images in *q*-space [6]:

16 $$\begin{aligned} C(q, \varDelta t) = 2 \tilde{T}(q) S(q) [1-f(q,\varDelta t)] + B(q) \end{aligned}$$

It contains an optical transfer function [36] $\tilde{T}(q) = 4 \sin ^2(q^2\,Z/(2k))$, a background term *B*(*q*), the static structure factor of the fluctuations *S*(*q*) and the intermediate scattering function $f(q,\varDelta t)$, that comprises a thermal and a solutal mode:

17 $$\begin{aligned} f(q,\varDelta t) = \frac{S^T(q)}{S(q)}\exp \left( -\frac{\varDelta t}{\tau ^T}\right) + \frac{S^c(q)}{S(q)}\exp \left( -\frac{\varDelta t}{\tau ^c}\right) \end{aligned}$$

The static structure factors of the thermal and the solutal NEFs add up to the total static structure factor $S(q) = S^T(q) + S^c(q)$. They diverge for large wavevectors proportional to $q^{-4}$,

18 $$\begin{aligned} S^i(q) = \frac{I_0^i}{1 + (q/q^i_{\mathrm{ro}})^4} ~~~~~~i=T,c ~, \end{aligned}$$

and they are cut off by gravity (gravitational acceleration *g*) below the respective roll-off wavevectors [43]

19 $$\begin{aligned} q^T_{\mathrm{ro}} = \left( \frac{\beta _T g \nabla T }{\nu D_{\mathrm{th}}}\right) ^{1/4} {\mathrm{and}}~~~ q^c_{\mathrm{ro}}= & {} \left( \frac{\beta _c g \nabla c }{\nu D}\right) ^{1/4} .\nonumber \\ \end{aligned}$$

The two time constants are given by [44]

20 $$\begin{aligned} \tau ^T(q)= & {} \frac{1}{D_{\mathrm{th}} q^2 (1 + (q^T_{\mathrm{ro}}/q)^4)} \end{aligned}$$

21 $$\begin{aligned} \tau ^c(q)= & {} \frac{1}{D q^2 (1 + (q^c_{\mathrm{ro}}/q)^4)} ~. \end{aligned}$$

The static structure factors and the time constants are determined from fits of the bimodal structure function (16) for fixed *q*-values.

The usual data evaluation is based on the assumption of a thin sample with constant concentration and temperature gradients throughout the liquid and with effectively constant values of all thermophysical parameters of the sample that correspond to the mean temperature in the center of the cell. In particular for systems with large Soret coefficients, such as polymer solutions or colloidal dispersions, and/or large temperature differences $\varDelta T > S_T^{-1}$, the concentration profile shows, however, a pronounced nonlinearity and $\nabla c$ is far from being constant. For the $30\,\hbox {K}$ temperature difference shown in Fig. 3, it increases by almost a factor of 20 from the hot to the cold plate.

Since the amplitudes of the NEFs (Eq. (18)) are proportional to the square of the respective gradient [2],

22 $$\begin{aligned} I_0^T \sim \frac{| \nabla T |^2}{\nu D_{\mathrm{th}}} ~~~ {\mathrm{and}} ~~~ I_0^c \sim \frac{| \nabla c |^2}{\nu D} ~, \end{aligned}$$

the signal of the solutal NEFs emerging from a thin layer near the cold wall in Fig. 3 will be about a factor of 400 stronger than the corresponding signal near the hot wall. Because of the nonlinear concentration profile, the measured signal will in particular not correspond to the conditions in the center of the cell. Since the time constants $\tau ^T(q)$ and $\tau ^c(q)$ also depend on $\nabla T$ and $\nabla c$ via the roll-off wavevectors (Eq. (19)), they will also vary from layer to layer. The temperature and concentration dependence of the optical contrast factors is generally only weak and has been neglected in Eq. (22).

#### Modeling of structure functions for thick samples

In order to understand how the experiments and the extracted data, e.g., diffusion and Soret coefficients, are affected, we will now simulate the expected multimodal measured signals. Then, we will apply the same standard data evaluation procedure as for real measurements, which is not aware of the nonlinearities, to these computed signals. The extracted coefficients can then be compared to the known input values and to shadowgraphy experiments.

We assume that the sample is composed of parallel horizontal slices with different values of temperature and concentration, for which Eqs. (16) to (22) hold individually. The total recorded signal $C(q, \varDelta t)$ is then computed as a linear superposition by averaging Eq. (16) over the cell height. For this purpose, we introduce the vertical coordinate *z* as an additional variable for all quantities that depend on *T* and *c* and, thus, on the position within the cell. Equation (16) now becomes

23 $$\begin{aligned} C(q,\varDelta t) = \frac{1}{h} \int _0^h 2 \tilde{T}(q,z) S(q,\varDelta t,z) \mathrm{d}z + B_0 ~. \end{aligned}$$

The local temperature *T*(*z*) and concentration *c*(*z*) are given by Eqs. (13) and (14), respectively. It should be noted that such an incoherent linear superposition [44] of the scattering from the different horizontal layers contains the approximation of uncorrelated fluctuations in the *z*-direction. Assuming that the vertical size of the fluctuations is as large as the horizontal one, we expect this assumption to be applicable as long as $q \gg 2 \pi /h \approx 12.6\,{\hbox {cm}^{-1}}$, which holds for almost all experimental wavevectors [45]. Besides the temperature and the concentration themselves and their respective gradients, the temperature and/or concentration dependence of the following quantities has been taken into account to calculate $C(q,\varDelta t)$ according to Eq. (23): the Soret coefficient $S_T$, the diffusion coefficient *D*, the thermal diffusivity $D_{\mathrm{th}}$, the kinematic viscosity $\nu$, and the density $\rho$. In addition, the optical transfer function $\tilde{T}(q)$ also depends weakly on *z* due to the different distances *Z* between the individual layers and the sensor.

**Fig. 4:**
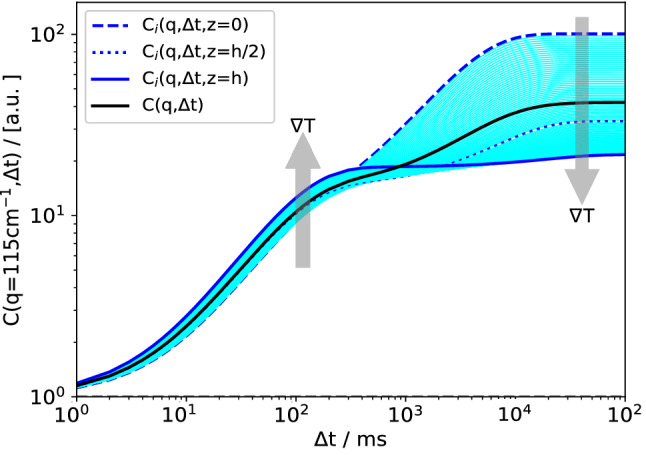
Simulated structure functions for different positions $0 \le z \le h$. The solid line is the average $C(q,\varDelta t)$ as observed in an hypothetical experiment with $q = 115\,{\hbox {cm}^{-1}}$. The dotted line corresponds to the center of the cell at $z=h/2$. PS($17.9\,{\hbox {kg/mol}}$)/Tol, $c_0 = 0.01$, $T_0 = 298.15\,\hbox {K}$, $\varDelta T = 30\,\hbox {K}$

Figure 4 shows $C(q,\varDelta t)$ for a fixed wave vector $q = 115\,{\hbox {cm}^{-1}}$ and a temperature difference of $\varDelta T = 30\,\hbox {K}$. Besides the experimentally observable mean structure function $C(q,\varDelta t)$ according to Eq. (23) (black solid line), also the structure functions $C_i(q,\varDelta t,z)$ for the individual layers are shown. As expected from the *z*-dependence of the concentration gradient shown in Fig. 3, the structure function with the largest amplitude originates from the layer at the cold window at $z = 0$ and the one with the lowest amplitude from the hot window at $z=h$. All structure functions for layers in between, i.e., for $0< z < h$, comprise the shaded region. The broad spreading of the solutal amplitudes by a factor of 30 is a consequence of both the temperature and concentration dependencies of the thermophysical parameters and of the nonlinearity of the diffusion equation (1). All thermal contributions, the fast modes, are confined within a narrow band with an amplitude variation by merely a factor of two. An important observation is that in particular, the observable averaged solutal structure function (thick solid line) is substantially different from the one that corresponds to the center of the cell ($C_i(q,\varDelta t,z=h/2)$, dotted line).

**Fig. 5:**
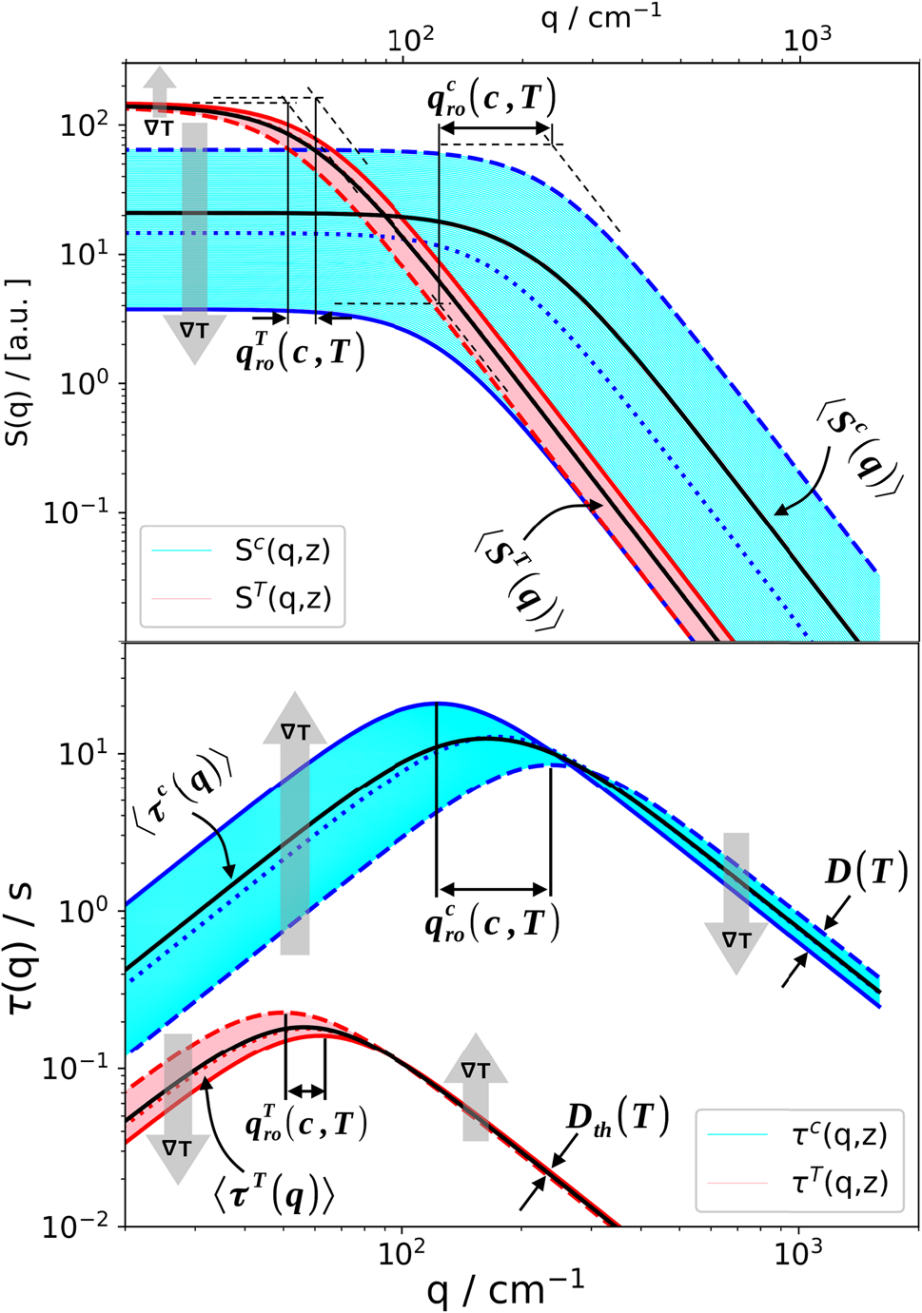
Variation of the amplitudes of the thermal and solutal contribution to the static structure factor (top) and thermal and solutal time constants (bottom) over the cell height from $z=0$ (cold, dashed lines) to $z=h$ (hot, solid lines). The dotted lines correspond to the center of the cell at $z=h/2$. Simulation PS($17.9\,\hbox {kg/mol}$)/Tol, $c_0 = 0.01$, $T_0 = 298.15\,\hbox {K}$, $\varDelta T = 30\,\hbox {K}$

Figure 5 shows how the static structure factors and the time constants vary over the height of the cell for the individual layers between $z=0$ and $z=h$. Again, the much broader dispersion of the solutal NEFs when compared to the thermal ones is evident. As shown in Fig. 5, also the roll-off wavevectors are different for the different layers.

#### Evaluation of simulated and experimental data

**Fig. 6:**
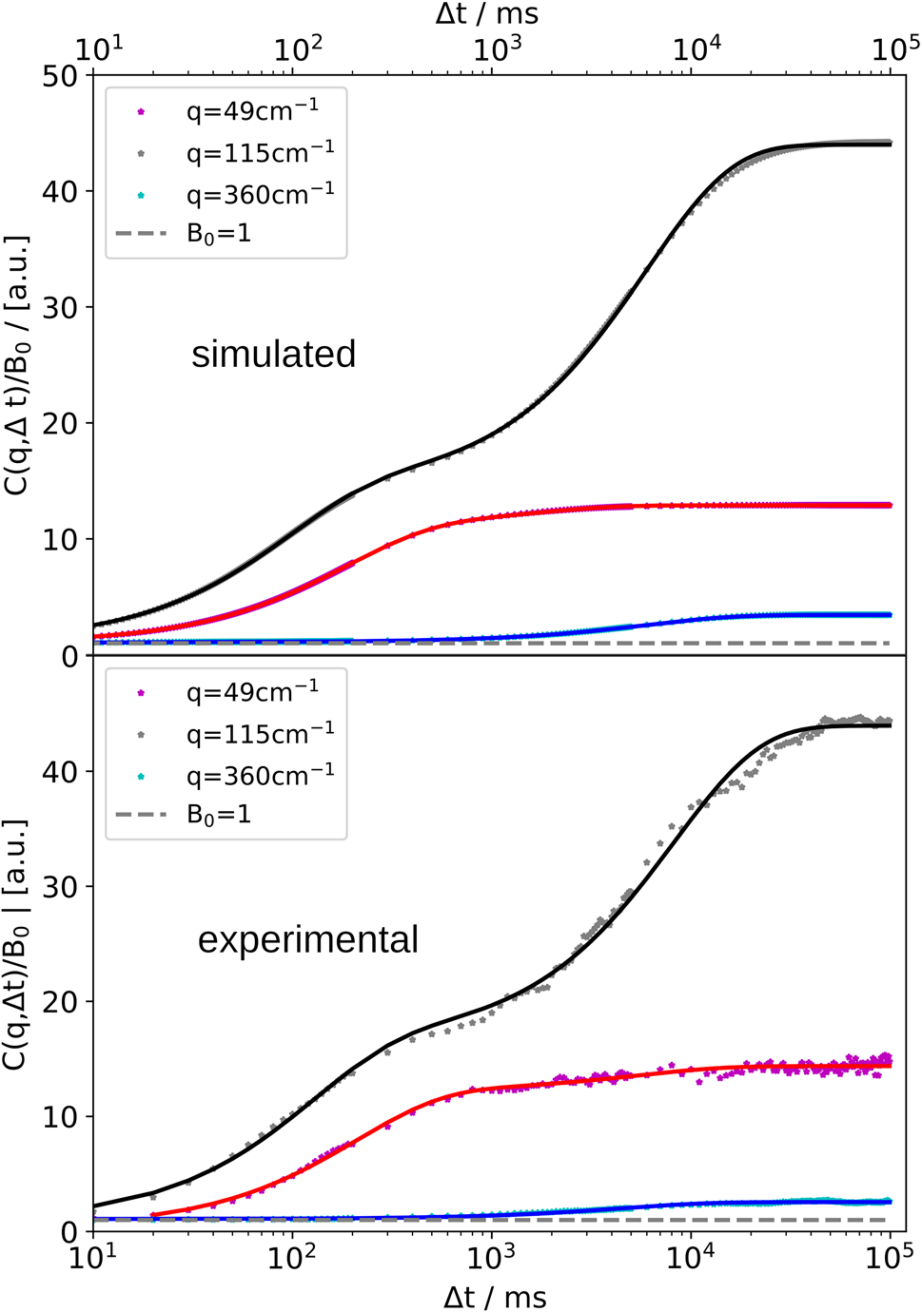
Fits of simulated averaged structure functions (top) and experimental structure functions (bottom) with bimodal structure factor from Eq. (16) for three different *q*-values. PS($17.9\,\hbox {kg/mol}$)/Tol, $c_0 = 0.01$, $T_0 = 298.15\,\hbox {K}$, $\varDelta T = 30\,\hbox {K}$

Once the simulated averaged structure functions $C(q,\varDelta t)$ are computed, they are treated in the same way as experimental correlation functions that are extracted from a time series of shadowgraph images. Thus, the next step is to fit the simulated averaged structure function (the black solid line in Fig. 4) with the bimodal structure function of Eqs. (16) and (17). The fits to both the simulated and the experimental data are shown in Fig. 6 for three *q*-values with an applied temperature difference of $\varDelta T = 30\,\hbox {K}$. Although the two contributions to the simulated structure functions are not single-exponential, due to the dispersion of the time constants $\tau ^c$ and $\tau ^T$, the fits match surprisingly well and hardly show any systematic deviation that could become visible in the presence of experimental noise.

**Fig. 7:**
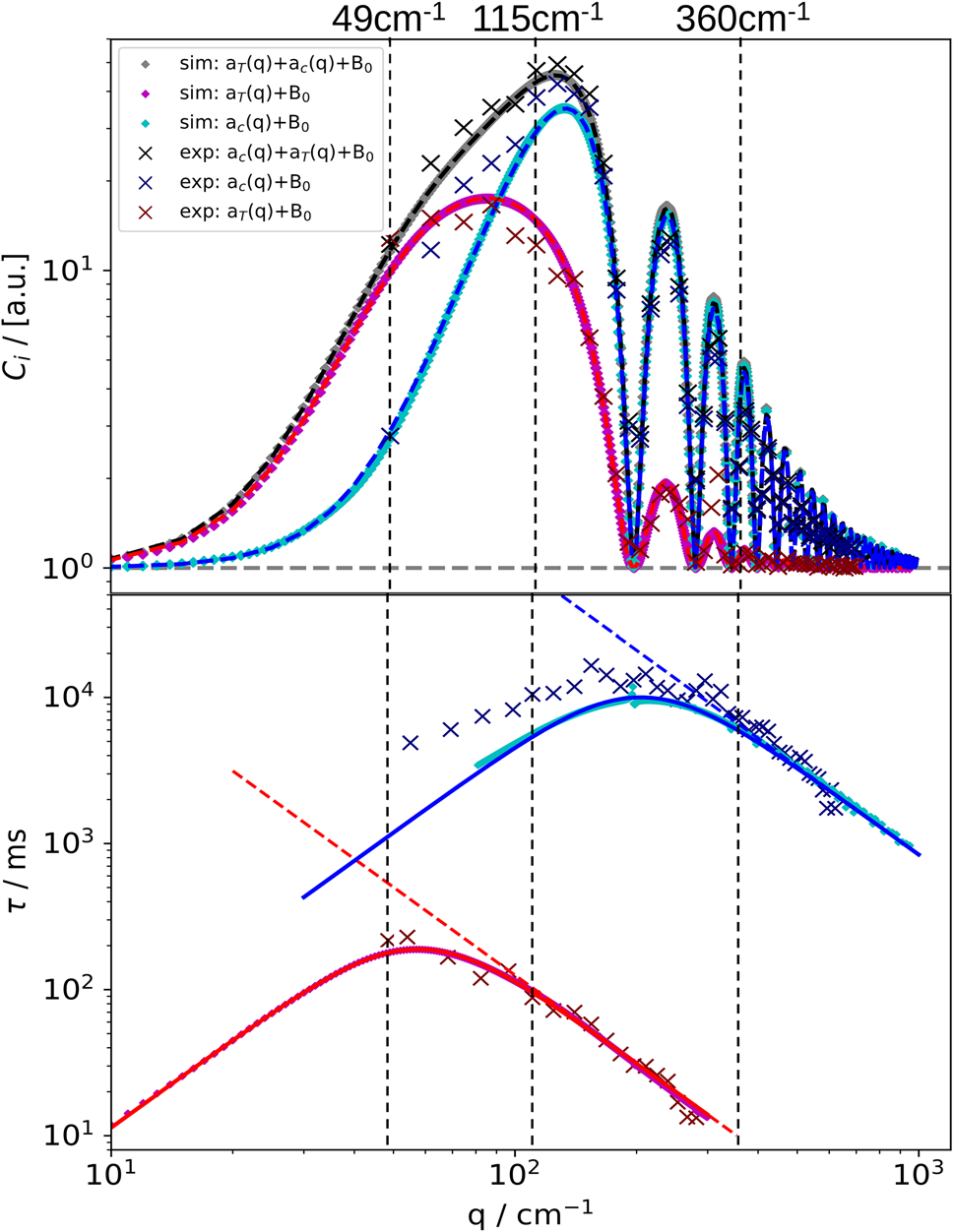
Results of the evaluation of the simulated structure functions (colored symbols) in comparison with experimental data (crosses). Top: Static amplitudes (thermal, solutal and total) of the the structure function $C(q,\varDelta t \rightarrow \infty )$. The dashed curves are fits of Eq. (16) to the simulated curves. Bottom: Thermal and solutal time constants $\tau ^T(q)$ and $\tau ^c(q)$ The solid lines are fits of Eqs. (20) and (21), respectively. The vertical dashed lines indicate the *q*-values of the structure functions in Fig. 6. PS($17.9\,\hbox {kg/mol}$)/Tol, $c_0 = 0.01$, $T_0 = 298.15\,\hbox {K}$, $\varDelta T = 30\,\hbox {K}$

The results of these fits that are performed for every single *q* are the amplitudes and the time constants of the thermal and the solutal mode, which are plotted in Fig. 7. The linear slopes on the high-*q* side yield the thermal diffusivity $D_{\mathrm{th}}$ and the diffusion coefficient *D*, respectively. On the low-*q* side, at *q*-values smaller than the positions of the maxima in the $\tau$-plots, the experimentally determined times systematically deviate from the simple model, as frequently observed in shadowgraphy experiments. Such deviations are to be expected, since the simulation does not account for additional effects that might become important for small *q*-vectors, e.g., the coupling of concentration, temperature and wall-normal velocity fluctuations [46]. These larger experimental times are also reflected in Fig. 6 for, e.g., $q = 115\,{\hbox {cm}^{-1}}$, where the solutal mode is shifted to somewhat longer times when compared to the simulation.

**Fig. 8:**
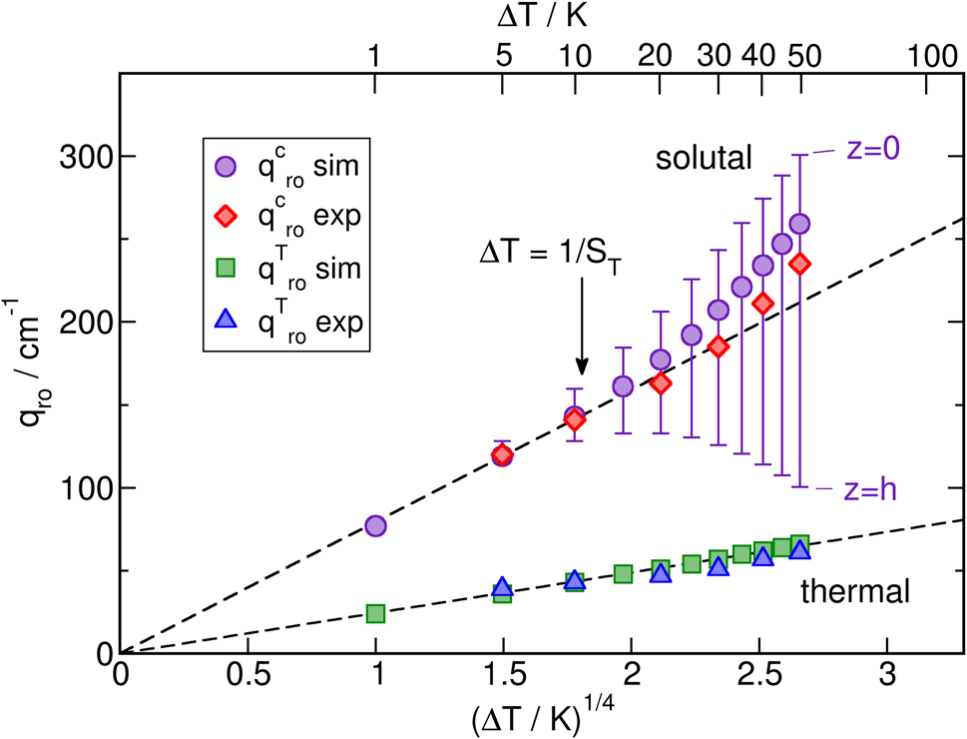
Thermal ($q^T_{\mathrm{ro}}$) and solutal ($q^c_{\mathrm{ro}}$) roll-off wavevectors as obtained from fits of the static structure factors of the simulated (sim) measurements and of the experimental (exp) data. The dashed lines correspond to the $q^{1/4}$-prediction of Eq. (19). The error-bar like vertical lines indicate the range of $q^c_{\mathrm{ro}}$ for the different layers. PS($17.9\,\hbox {kg/mol}$)/Tol, $c_0 = 0.01$, $T_0 = 298.15\,\hbox {K}$, $\varDelta T = 30\,\hbox {K}$

The roll-off wavevectors $q_{\mathrm{ro}}^c$ and $q_{\mathrm{ro}}^T$ are determined from a simultaneous fit of Eqs. (16) to (18) to the *q*-dependence of the static amplitudes as obtained from the time-dependent fits of the structure functions (Fig. 7). They are plotted in Fig. 8 both for the simulation and the experiment. The agreement is reasonable albeit not perfect.

As predicted by Eq. (19), the plot of $q_{\mathrm{ro}}^T$ vs. $\varDelta T^{1/4}$ is nicely linear over the entire temperature gradient range. The dashed line is the prediction from Eq. (19) for $T_0 = 298.15\,\hbox {K}$ without taking any variation of parameters across the cell height into account.

The solutal counterpart $q_{\mathrm{ro}}^c$ follows Eq. (19) only for small temperature differences $\varDelta T \lesssim 10\,\hbox {K}$, as shown by the linear fit to the lowest three simulated data points. For larger temperature differences, both the simulated and the experimental data increasingly deviate toward larger wavevectors. Equation (2) yields a Soret coefficient of $S_T = 0.092\,{\hbox {K}^{-1}}$. The deviation from the linear fit becomes significant for $\varDelta T > S_T^{-1} \approx 10\,\hbox {K}$, which is where the concentration profile inside the cell starts to become nonlinear. The vertical lines in Fig. 8, which resemble error bars, indicate the range over which $q_{\mathrm{ro}}^c$ varies from layer to layer between $z=0$ and $z=h$. Remarkably, the simulated and measured $q_{\mathrm{ro}}^c$ are significantly shifted from their initial center position for $\varDelta T > S_T^{-1}$ toward the cold layer at $z=0$.

Finally, the Soret coefficient is calculated from the roll-off wavevectors according to [16]

24 $$\begin{aligned} S_T = \frac{-1}{c(1-c)} \frac{\beta _T}{\beta _c} \frac{D}{D_{\mathrm{th}}} \left( \frac{q_{\mathrm{ro}}^c}{q_{\mathrm{ro}}^T}\right) ^4~. \end{aligned}$$

Unfortunately, the dependence on the fourth power of the roll-off wave vectors leads to a rather unfavorable error-amplification. As an alternative approach, the temperature gradient can be taken directly from the applied temperature difference instead of the thermal roll-off wavevector [8]. For the experiments reported here, the difference is negligible, as can be seen from the very good agreement of the simulated and experimental values of $q_{\mathrm{ro}}^T$ in Fig. 8.

**Fig. 9:**
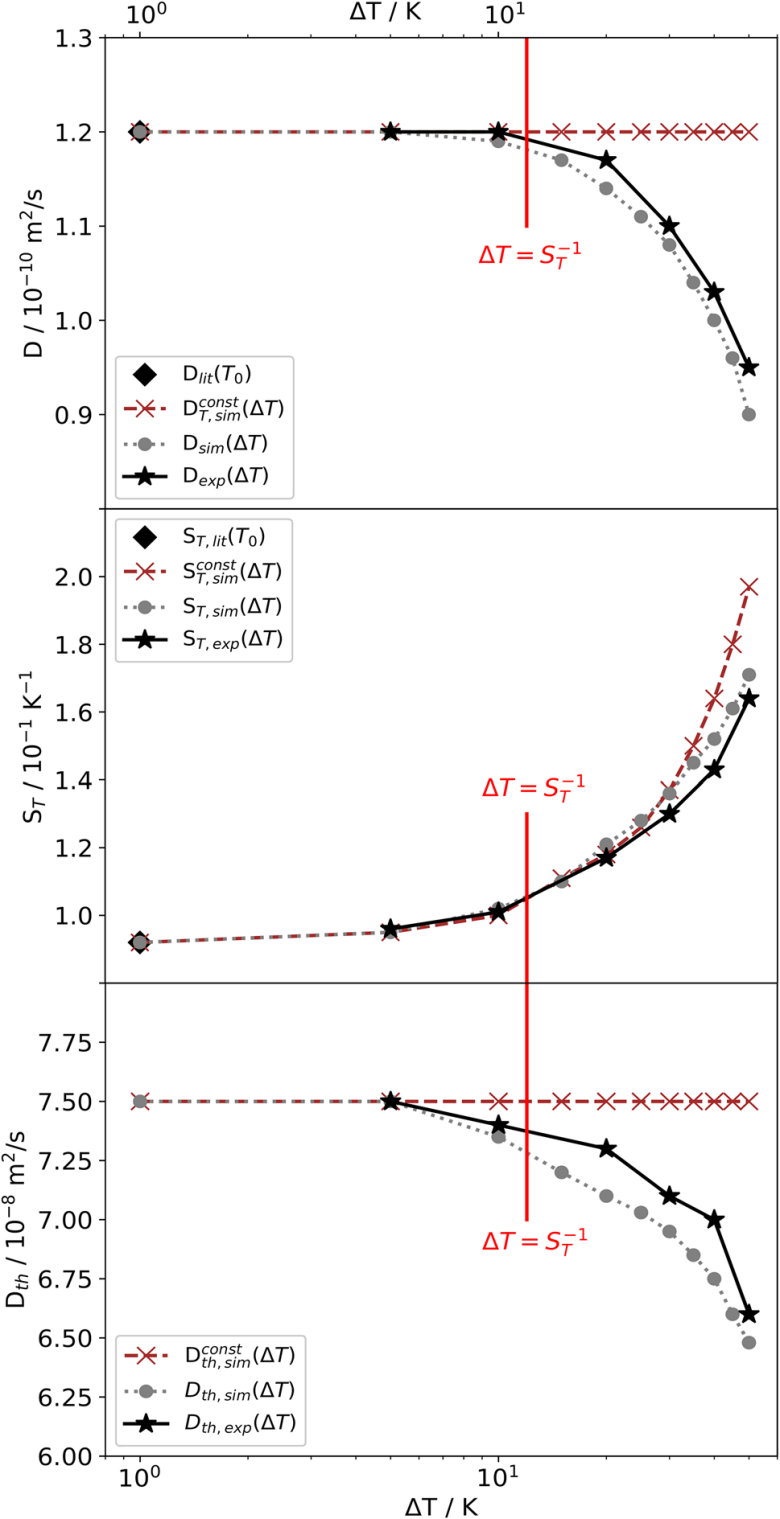
Measured (exp) and simulated (sim) diffusion coefficients *D* (top), Soret coefficients $S_T$ (middle) and thermal diffusivities $D_{\mathrm{th}}$ (bottom) as a function of the applied temperature difference $\varDelta T$. The literature values (lit) are directly computed from Eqs. (2) and (3) and coincide with the simulated values for $\varDelta T \rightarrow 0$. Also shown are simulations performed for the case of temperature and concentration independent thermophysical parameters (labeled as *const*). PS($17.9\,\hbox {kg/mol}$)/Tol, $c_0 = 0.01$, $T_0 = 298.15\,\hbox {K}$, $\varDelta T = 30\,\hbox {K}$

Figure 9 shows $D_{\mathrm{th}}$, *D*, and $S_T$ as obtained from the evaluation of the simulated structure functions in comparison with the experimental values. Up to $\varDelta T \approx S_T^{-1}$ all three coefficients agree rather well with their nominal values, but beyond they deviate significantly. In particular, the apparent Soret coefficient deviates by a factor of two from its true value at $T=T_0$ and $c=c_0$ for $\varDelta T = 50\,\hbox {K}$. The deviations of the diffusion coefficient and the thermal diffusivity are less pronounced and amount to approximately 25 and 12 percent, respectively.

Also shown in Fig. 9 are the coefficients that have been obtained from a simulation that neglects all temperature and concentration dependencies and only takes the nonlinearity of the concentration profile into account. As expected, the $\varDelta T$-dependence of the measured $D_{\mathrm{th}}$ and *D* results only from the temperature and concentration dependencies and not from the nonlinearity of the diffusion equation. The deviation of $S_T$ for larger $\varDelta T$, on the other hand, follows mainly from the nonlinearity of the concentration profile and is even weakened by the temperature and concentration dependencies of the parameters. As for $q_{\mathrm{ro}}^c$ in Fig. 8, the measured and simulated coefficients are only correct for sufficiently small $\varDelta T < S_T^{-1}$.

## Summary and conclusions

We have investigated a problem that is inherent to many nonequilibrium experiments, which require finite temperature and/or concentration gradients in order to generate a measurable signal. The results are then typically assigned to a fixed temperature, which frequently corresponds to the mean sample temperature. Whether this approach is valid or not cannot easily be predicted. It depends on the temperature dependence of all relevant physicochemical parameters. For the polymer solution studied here, the most crucial question is, whether the underlying transport equations, the heat equation and the extended diffusion equation, can be linearized with respect to the temperature and concentration gradients. While this assumption is generally fulfilled for the temperature gradient, which is approximately constant even for large temperature differences of say $\varDelta T = 50\,\hbox {K}$, the concentration gradient develops a pronounced nonlinearity already for much smaller temperature differences. The major reason for the nonlinear concentration gradient is the nonlinear term in the extended diffusion Eq. (1).

We have modeled the measured shadowgraphy signal as a superposition of the signals from parallel thin fluid layers, each of which characterized by its own temperature and concentration, and the respective gradients. Even though both the amplitudes of the concentration mode and the corresponding relaxation time constants vary over a broad range, as shown in Figs. 4 and 5, the superposition of the individual structure functions looks very ordinary and does not contain an apparent hint to the nonlinearity. It still can be approximated almost perfectly by the double-exponential fit of Eq. (17). The amplitudes, time constants and roll-off wavevectors that are extracted from these fits differ, however, significantly from the values expected from a linear model. In our example, this has led to significantly underestimated diffusion coefficients and to Soret coefficients that are overestimated by almost a factor of two for a temperature difference of $50\,\hbox {K}$.

We have compared our simulations to experimental shadowgraphy results for the diffusion and Soret coefficient as measured with different temperature gradients and found good agreement. It is difficult to generalize our findings, but it can be said that the signal anomaly mainly results from both the nonlinear concentration profile and from the temperature and concentration dependence of the system parameters. Thus, as a rule of thumb, the temperature difference should not exceed the inverse Soret coefficient: $\varDelta T < S_T^{-1}$. In particular for dilute solutions of high polymers [29], where the Soret coefficient can easily exceed $1\,{\hbox {K}^{-1}}$, this requirement can lead to severe experimental limitations that require careful consideration. In our example in Fig. 9, the apparent Soret coefficients deviate toward larger values, which makes the criterion $\varDelta T < S_T^{-1}$ a safe one when checked against the experimentally determined Soret coefficient. Such a good-natured overestimation is, however, not guaranteed. The direction depends on details of the experiment and on the concentration and temperature dependence of $S_T(c,T)$. This might quite as well lead to an unfavorable underestimation of the Soret coefficient when measured with too large temperature gradients.

Our results are a contribution to the Giant Fluctuations and the TechNES projects of the European Space Agency ESA and the BTGIANT project of the German Aerospace Center DLR, which aim at the understanding of NEFs in non-ideal systems as encountered in the presence of large gradients both on ground and in microgravity.

## Data Availability

Data are available upon reasonable request from the authors.
